# Improving soil water-salt conditions and cotton yield through optimized C/N fertilizer ratios and irrigation in arid regions

**DOI:** 10.3389/fpls.2026.1848146

**Published:** 2026-06-03

**Authors:** Kexin Liu, Shi Wang, Hao Zhang, Baiqing Chen, Bingli Wang, Shaoyu Cheng, Qiuxiang Tang, Fengquan Wu

**Affiliations:** 1 College of Agronomy, Engineering Research Centre of Cotton, Xinjiang Agricultural University, Ürümqi, Xinjiang, China; 2 Institute of Cotton Research, Chinese Academy of Agricultural Sciences, Anyang, China

**Keywords:** C/N ratio, irrigation amounts, residual film contamination, soil moisture, soil salinity, yield

## Abstract

**Introduction:**

Plastic mulch is widely used in agriculture as it increases soil temperature and reduces water evaporation, thereby enhancing water use efficiency and crop yields. However, prolonged use of plastic mulch leaves residues that negatively impact soil structure, impede water and salt movement, and reduce cotton yields. While the impact of mulch contamination on water-salt distribution is recognized, the specific mechanism by which increasing the C/N ratio improves this distribution remains unclear. We hypothesize that by raising the C/N ratio, it is possible to enhance the water-salt environment, thus stabilizing lint cotton yields.

**Methods:**

A two-year field experiment (2024–2025) was conducted in Xinjiang, China. Three irrigation levels were established based on crop evapotranspiration (ETc): 0.6 ETc (I1, severe deficit), 0.8 ETc (I2, mild deficit), and 1.0 ETc (I3, full). Four C/N ratio treatments were applied: 0:1 (F0), 3:1 (F1), 6:1 (F2), and 9:1 (F3).

**Results:**

Across irrigation levels, increasing the C/N ratio of the fertilizer significantly enhanced soil water-holding capacity, reduced salt accumulation, and improved water and salt uniformity. Yield under C/N 6:1 (F2) was 7.9% higher on average than other treatments. Under different C/N ratios, mild deficit irrigation (I2) maintained higher soil moisture uniformity and desalination rate(Rs); soil salinity was 5.4% lower than under I1 but 9.5% higher than under I3; seed cotton yield was 17.0% and 0.8% higher than I1 and I3, respectively. Compared with I3F0, I2F2 increased soil moisture by 4.2%, moisture uniformity by 4.1%, reduced salinity by 1.1%, and raised yield by 9.1%. Correlation analysis showed desalination rate, moisture content, and its uniformity coefficient were significantly positively correlated with lint yield, while soil salinity was significantly negatively correlated.

**Discussion:**

A C/N ratio of 6:1 effectively improves the water-salt balance in plastic mulch-contaminated cotton fields by optimizing soil water and salt distribution, thereby increasing yield. This provides mechanistic insight and practical strategies for water-saving and salt-suppression management in arid regions.

## 1 Introduction

Cotton(*Gossypium hirsutum L.*)stands as one of the world’s most important cash crops (Zhang et al., 2022a) and is primarily cultivated in the Xinjiang region of China. In recent years, with the widespread adoption of plastic mulch technology, both the area under cotton cultivation and cotton yields in Xinjiang have continued to grow; by 2024, the region’s output accounted for more than 91.4% of the national total (Lu et al., 2024)。Nevertheless, the ultra-thin polyethylene (PE) films commonly used in agriculture, with a thickness of just 4-8 μm, pose significant challenges for complete recycling (Schyns and Shaver, 2021).These film residues are often burned, dumped, buried, or piled up in fields (Dong et al., 2022), As they accumulate in the soil over the long term, they not only alter soil water and salt transport characteristics (Adeyemo et al., 2022; Zhang et al., 2022b), but also suppress root water uptake (Gu et al., 2024), leading to reduced cotton yields (Cao et al., 2022),leading to reduced cotton yields (Chen et al., 2022; Hu et al., 2026). Therefore, against the backdrop of film residue pollution, improving the soil water and salt environment is of great significance for maintaining sustainable cotton production (Zhao et al., 2023).

Adjusting the soil carbon-to-nitrogen (C/N) ratio is a sustainable agronomic practice that can improve soil properties and reduce environmental risks by enhancing organic matter cycling and optimizing microbial community structure (Zhang et al., 2020; Li et al., 2026). Long-term use of chemical fertilizers can easily affect soil water and salt movement, leading to salinization, acidification, and the loss of organic matter, thereby impacting crop growth (Wu et al., 2020). Existing studies have shown that regulating the soil C/N ratio can promote the retention of soil moisture and nutrients (Xu et al., 2026, 2025), increase crop yield (Li et al., 2022b), and reduce soil salt content (Wang et al., 2023). Under conditions of relatively ample nitrogen, a low C/N ratio promotes the rapid decomposition of organic matter and nitrogen mineralization. In the short term, this increases the supply of available nitrogen in the soil, stimulates cotton root growth and nutrient uptake, and thereby alleviates nutrient limitations caused by water stress to some extent (Chaves et al., 2021). Therefore, it is crucial to scientifically determine an appropriate C/N ratio in conjunction with soil water, salt, and nutrient conditions (Chen et al., 2025a). Previous research has also indicated that applying C/N ratios under mild irrigation can significantly improve water use efficiency and increase yield (Zhang et al., 2024b). Thus, in arid and water-scarce regions, regulating the C/N ratio under deficit irrigation conditions can improve the soil water and salt environment (Wang et al., 2024) while achieving water-saving and yield-stabilizing effects (Li et al., 2024b; Niu et al., 2024).

Irrigation management is one of the key measures for improving the water and salt environment in the crop root zone (Li et al., 2024a). Under saline soil conditions in arid areas, appropriate deficit irrigation can promote salt leaching and reduce salt accumulation while saving water (Guo et al., 2025), and effectively enhance crop yield (Wang et al., 2024). However, excessive deficit irrigation can weaken the uniformity of water and salt distribution, restrict the downward migration of salts, and lead to an increase in the salt concentration of the soil solution in the root zone (Yang et al., 2024; Li et al., 2022a). A high-salinity environment can trigger multiple adverse effects, including osmotic stress, ion toxicity, and nutrient imbalance, thereby inhibiting crop root growth and photosynthetic metabolism, and ultimately causing a significant yield decline (Ahmad et al., 2023). Therefore, when optimizing irrigation quotas based on crop water requirements, a balance must be struck between water-saving benefits and salt-suppression effects to maintain a favorable root-zone water and salt environment and ensure high and stable yields.

Adjusting the C/N ratio can reduce the direct toxicity of salt ions to crops while enhancing soil water-holding capacity, mitigating drought damage, and stabilizing crop yields by conserving water and maintaining soil moisture (Chen et al., 2023; Zhang et al., 2024a; Elmeknassi et al., 2024). Adjusting the C/N ratio can effectively mitigate the uneven distribution of water and salts caused by deficit irrigation, as well as the impeded downward migration of salts (Liu et al., 2025). However, under conditions of plastic film residue contamination in arid and water-scarce regions, the coupling mechanism between the C/N ratio—a conventional practice for altering soil properties—and irrigation, which drives water and salt movement, and their interaction, on soil water and salt distribution and cotton yield remains unclear. Based on this, this study hypothesizes that in cotton fields contaminated by plastic film residues, an appropriate C/N ratio can improve the water and salt environment in the root zone, enhance the uniformity of water and salt distribution, and reduce the degree of salinization, thereby mitigating the adverse effects of deficit irrigation and promoting the stability and increase of cotton yield. To test this hypothesis, we conducted a two-year field experiment in typical plastic film-contaminated cotton fields in Xinjiang, with the primary objectives being: (a) To evaluate the effects of different irrigation levels and C/N application ratios on cotton yield in fields contaminated by plastic mulch residues; (b) To clarify their roles in improving the uniformity of soil water and salt distribution and the desalinization effect in such fields; (c) To elucidate the relationship between soil water and salt characteristics and yield, thereby providing a scientific basis for water-saving, salt-suppression, and yield-stabilization management in areas contaminated by plastic mulch residues.

## 2 Materials and methods

### 2.1 Overview of the pilot zone

**Figure 1:**
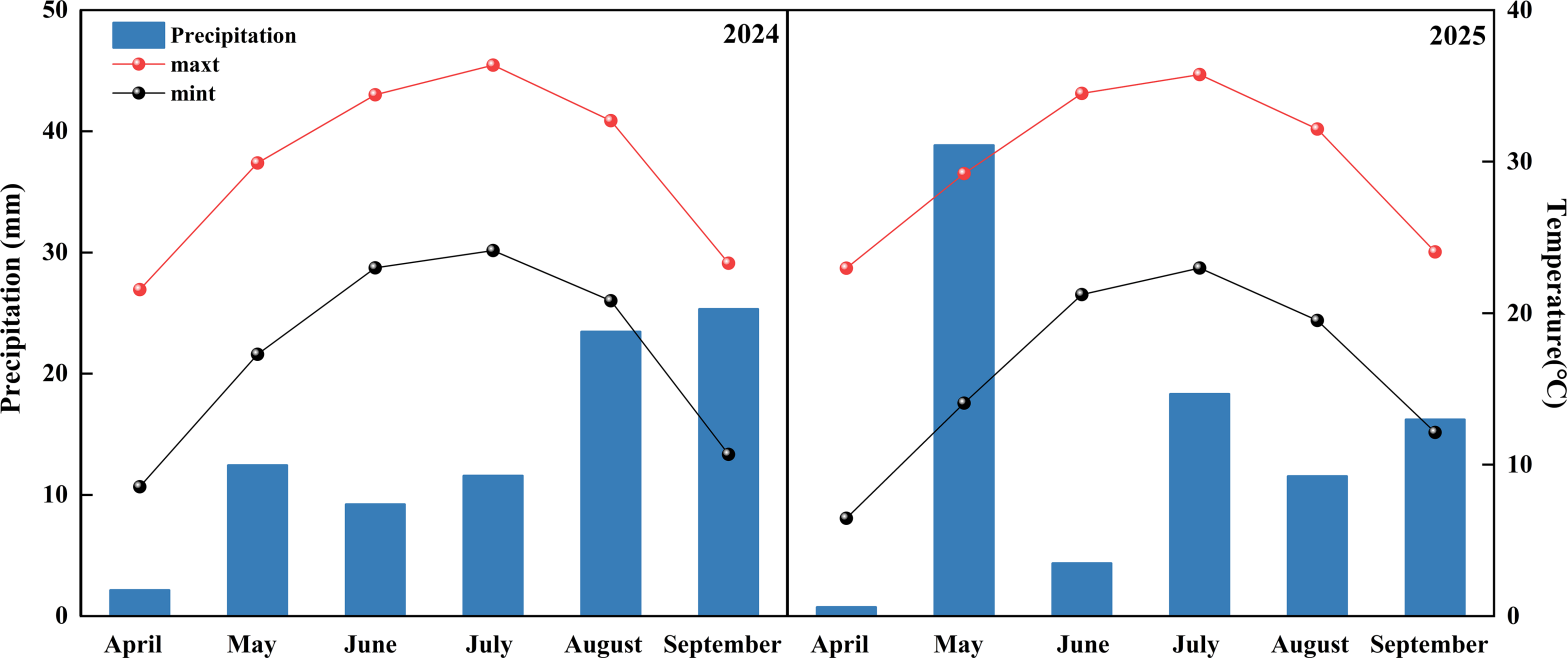
Temperature and precipitation during the cotton growing season.

A two-year field experiment was conducted from 2024 to 2025 at the study area of the Western Agricultural Research Center of the Chinese Academy of Agricultural Sciences (44°15’N, 87°19’E) in Xinjiang, China, in a cotton field with typical plastic mulch residues contamination. The region has a typical temperate continental arid climate, with an average annual temperature of 6.8 °C and approximately 170 days per year with accumulated temperatures exceeding 10 °C. Annual total sunshine hours are 2,700 hours, and the annual frost-free period ranges from 160 to 190 days. Annual precipitation averages 190 mm. The area consists of long-term plastic-mulched cotton fields; the soil in the test area is gray desert soil. In 2024, the plastic mulch residue was 254.50 kg hm^-2^ (**Table 1**), significantly higher than the typical plastic mulch residue in China of 75 kg hm^-2^ (Yan et al., 2014). The soil pH was 7.98, with organic matter at 14.61 g·kg^-1^, total N at 0.65 g·kg^-1^, available phosphorus at 22.04 mg·kg^-1^, and available potassium at 296.00 mg·kg^-1^. Maximum and minimum temperatures and precipitation during the period from April to October 2024-2025 (the crop growing season) (**Figure 1**). These data were obtained from an automatic weather station (Watch Dog 2900ET Weather Station, Spectrum, Inc., Plano, TX, USA) located near the experimental station.

**Table 1:** Distribution of residual plastic film at different soil depths before sowing in 2024 and after harvest in 2025 (kg hm^-2^).

Soil depth	2024		2025	
	Weight	Percentage	Weight	Percentage
(cm)	(kg·hm^-2^)	(%)	(kg·hm^-2^)	(%)
0-20	180.02	70.70	185.49	70.23
20-40	74.49	29.30	78.63	29.77
Total	254.51	100.00	264.12	100.00

### 2.2 Experimental design

The experiment employed a split-plot design, with the main plots consisting of three irrigation levels: severe water deficit at 0.6 ET_c_ (I1), mild water deficit at 0.8 ET_c_ (I2), and full irrigation at 1.0 ET_c_ (I3); The sub-plots comprised four C/N ratios: 0:1 (F0), 3:1 (F1), 6:1 (F2), and 9:1 (F3), with corresponding fertilizer application rates based on the C/N ratios (**Table 2**). The organic carbon source was matured cattle manure (application rates were calculated based on N content converted to pure N), the organic carbon content of the matured cattle manure was 36.51%, N 3.26%, P_2_O_5_1.28%, K_2_O 2.27%, C:N = 12:1, N sources included urea (N 46.8%, C:N = 1:2) and the organic N content of the matured cattle manure; the matured cattle manure, phosphorus, potassium, and 20% of the chemical fertilizer N were applied as base fertilizer; the remaining 80% of the chemical fertilizer N was divided into 10 equal portions and applied with irrigation water.

**Table 2:** Fertilizer application rates for different C/N ratios.

Treatment	C:N	Organic carbon	Total N/(kg·hm^-2^)		
		(kg·hm^-2^)	Organic fertilizer	Inorganic fertilizer	Total
F0	0:1	0	0	375	375
F1	3:1	1125	93.75	281.25	375
F2	6:1	2250	187.5	187.5	375
F3	9:1	3375	281.25	93.75	375

In this case, *ET_c_* is calculated using the following formula (Marin et al., 2019), as shown in Equation 1:

(1) $$ET_{c}=ET_{0\;}\times K_{c}$$

In this case, *ET*_0_ is calculated using the following formula (Allen et al., 1998), as shown in Equation 2:

(2) $$ET_{0}=\frac{0.408_{\Delta }(R_{n}-G)+\gamma \frac{900}{T_{mean}+273}u_{2}(e_{s}-e_{a})}{\Delta +\gamma (1+0.34u_{2})}$$

Where *ET*_0_ is the reference evapotranspiration (mmday^-1^); Δ is the slope of the saturation vapor pressure curve at air temperature (kPa°C^-1^); *R_n_* is the net radiation at the crop surface (MJm^-2^d^-1^) G is the soil heat flux density (MJm^-2^d^-1^); γ is the psychometric constant = 0.665× 10^-3^×P(kPa°C^-1^); P is the atmospheric pressure (kPa); *u_2_* is the wind speed at a 2 m height (ms^-1^); *e_s_* is the saturation vapor pressure (kPa); *e_a_* is the actual vapor pressure (kPa); (*e_s_*-*e_a_*) is the saturation vapor pressure deficit (kPa); and *T*_mean_ is the daily air temperature at a 2 m height (°C).

Each experimental plot measured 57 m^2^ (10.0 m long by 5.7 m wide) High-yield upland cotton (variety Zhongmian 113) was sown in early April and harvested in early October. Sub-mulch drip irrigation was employed, with the drip tape placed beneath the plastic mulch. The irrigation water level was controlled via solenoid valves and flow meters. To prevent the edge effect of water flow between plots, a 50-cm-wide trench was dug along the boundary of each plot and lined with polyvinyl chloride (PVC). The plastic mulch, which was 2.05 m wide and covered three crop rows, achieved a coverage rate of 81%. Drip tapes were laid out in rows with 25 cm spacing between emitters, each emitting at a flow rate of 1.6 L h^-1^. Over the two-year period, all plots were irrigated and fertilized once every 7 days, with the first irrigation on June 5 and the final irrigation on August 17. All other field management practices were consistent with local field production standards.

### 2.3 Test items and methods

#### 2.3.1 Soil water content

During key growth stages of cotton (29, 49, 73, 102, and 136 days after emergence), soil samples were collected from the 0–60 cm depth in both the wide-row and narrow-row plots within each treatment using a soil auger, with one layer collected every 10 cm. The samples were analyzed using the oven-drying method (Immanuel and Suriya, 2024).

#### 2.3.2 Soil salinity content

Total soil salinity was determined using the conductivity method, and soil samples were collected using the same method as for soil moisture measurement. Soil samples were dried, ground, sieved (using a 1 mm sieve), and impurities were removed. An extract was then prepared using a soil-to-water ratio of 5:1. Conductivity values were measured using a DDS-307 conductivity meter. Total dissolved solids (TDS) were then calculated based on the fitted relationship between conductivity and salt content (Ismayilov et al., 2021).

#### 2.3.3 Christiansen uniformity coefficient

The uniformity of soil moisture content and salinity distribution can be expressed using the Christiansen uniformity coefficient (C*_u_*) (Green and Pattison, 2022), as shown in Equation 3:

(3) $$C_{u}=1-\frac{\sum_{i=1}^{n}|X_{i}-\bar{X}|}{n\bar{X}}$$

In Equation 3: C*_u_* is the Christiansen uniformity coefficient (%); X_i_ is the salinity of the i-th soil layer (g·kg^-1^); x is the mean soil salinity (g·kg^-1^); n is the number of samples.

#### 2.3.4 Desalination rate

The desalination rate is a measure of desalination rate; it is calculated as shown in Equation 4 (Yin et al., 2022):

(4) $$R_{s}=\frac{\left(S_{1}-S_{2}\right)}{S_{1}}$$

In Equation 4: R_S_ is the desalination rate (%); S_1_ is the soil salinity before sowing (g·kg^-1^); S_2_ is the soil salinity after harvest (g·kg^-1^).

#### 2.3.5 Total dry matter accumulation

During the harvest period, five representative plants were randomly selected from each plot. The plants were initially dried at 105 °C for 1 hour to inactivate enzymes and fix tissue color, and then at 75 °C until constant weight to determine the total dry matter accumulation (DMA) per plant. The above-ground DMA per unit area (kg·ha^-1^) was calculated by multiplying the mean per-plant DMA by the average planting density of 237,000 plants·ha^-1^.

#### 2.3.6 Seed cotton yield and harvest index

During the cotton harvesting period, three randomly selected plots of 2.3 m × 1 m in length × width were hand-harvested. Then 100-boll weight, plant number and boll number were determined to calculate the seed cotton yield(Y).

The harvest index (HI) was measured by the ratio of seed cotton yield and total dry matter accumulation (Hu et al., 2025).

(5) $$HI=\frac{Y}{DMA}$$

In Equation 5, Y is the cotton seed yield (kg hm^-2^), and DMA is the total dry matter accumulation (kg hm^-^²).

### 2.4 Data processing

The experimental data from the three growing seasons were analyzed using analysis of variance in SPSS 22.0(SPSS Inc., Chicago, IL, USA). The data obtained from each sampling event were analyzed separately. When a significant treatment effect was observed at P< 0.05, the least significant difference (LSD) was used to determine differences between means. ANOVA was conducted using the year (Y), irrigation amount (I), and fertilization treatment(F) as the primary effects, and interactions included I×F and Y×I×F. Origin 2019b software (Origin Lab, Northampton, USA) was used to generate the figures.

## 3 Results and analysis

### 3.1 Soil moisture content and distribution

**Figure 2:**
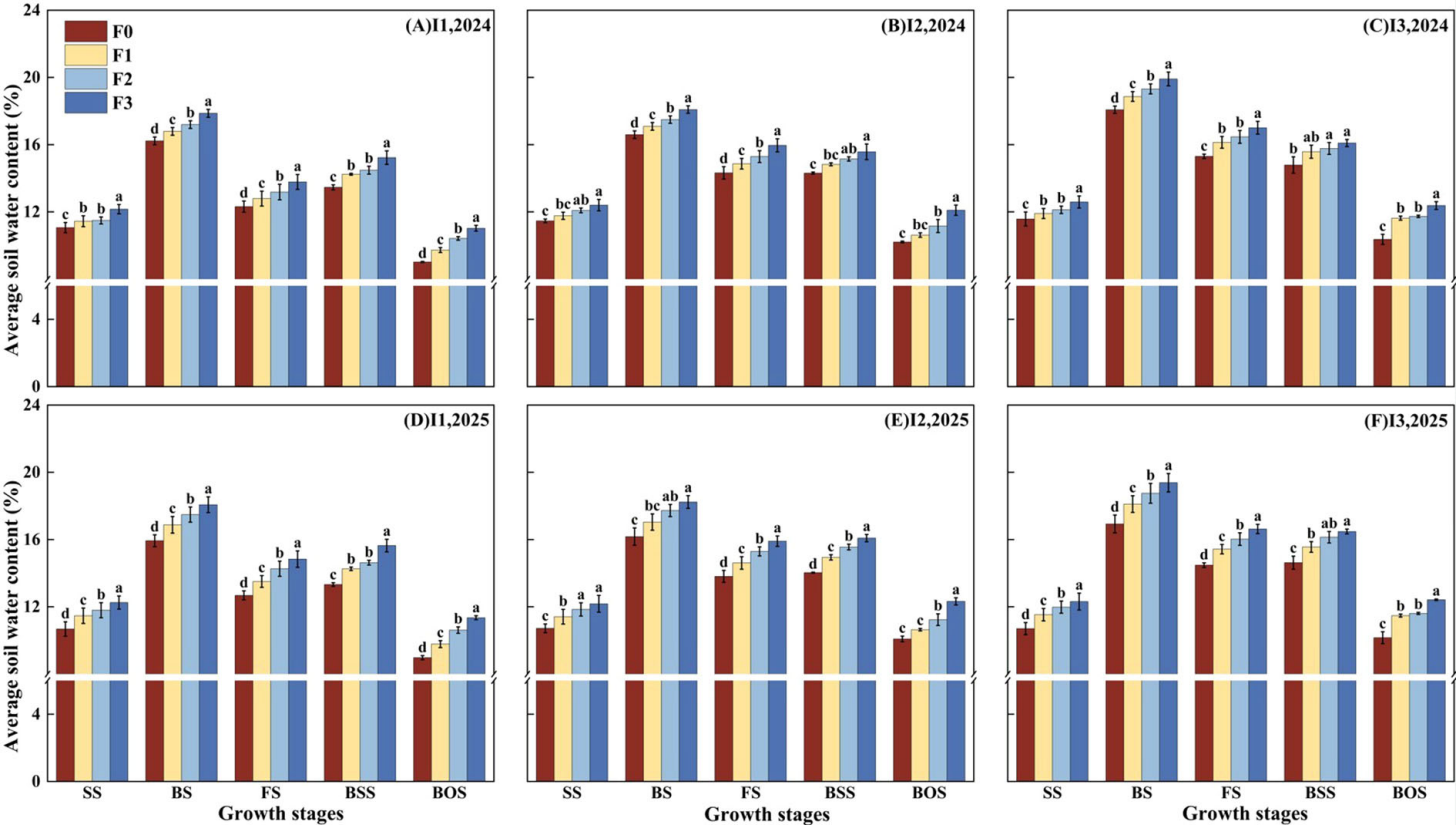
Average soil moisture content (0–60 cm) in cotton fields contaminated with pesticide residues under different irrigation levels and C/N ratios. The error bars represent the standard error of three replicates. Values with different lowercase letters are significantly different at P<0.05 using the LSD method. The average irrigation levels for I1, I2, and I3 were 0.6 ET_c_, 0.8 ET_c_, and 1.0 ET_c_, respectively. The C/N ratios for F0, F1, F2, and F3 were 0:1, 3:1, 6:1, and 9:1, respectively. SS denotes the seedling stage, BS denotes the budding stage, FS denotes the flowering stage, BSS denotes the boll stage, and BOS denotes the boll opening stage. **(A–C)** and **(D–F)** represent different irrigation treatments (I1, I2, and I3) in 2024 and 2025, respectively.

Increasing the C/N ratio significantly improved soil water content (SWC) in cotton fields contaminated with plastic film residues (**Figure 2**). The two-year study showed that under severely water-deficient irrigation (I1) conditions, the SWC for the 9:1 C/N ratio treatment (F3) was 4.9%-15.0% higher than that of other C/N treatments; under mild water-deficient irrigation (I2), F3 increased SWC by 4.2%-13.0% compared to other C/N treatments; and under full irrigation (I3), F3 increased SWC by 3.5%-13.3% compared to other C/N treatments. Under the combined effects of C/N ratio and irrigation volume, SWC under mild irrigation deficit plus a C/N ratio of 6:1 (I2F2) increased by 4.2% compared to full irrigation plus a C/N ratio of 0:1 (I3F0), but decreased by 8.0% compared to the fully irrigated treatment with a C/N ratio of 9:1 (I3F3). In summary, increasing the C/N ratio can compensate for water deficits caused by irrigation deficiency and improve soil moisture content in cotton fields contaminated by plastic film residues.

**Figure 3:**
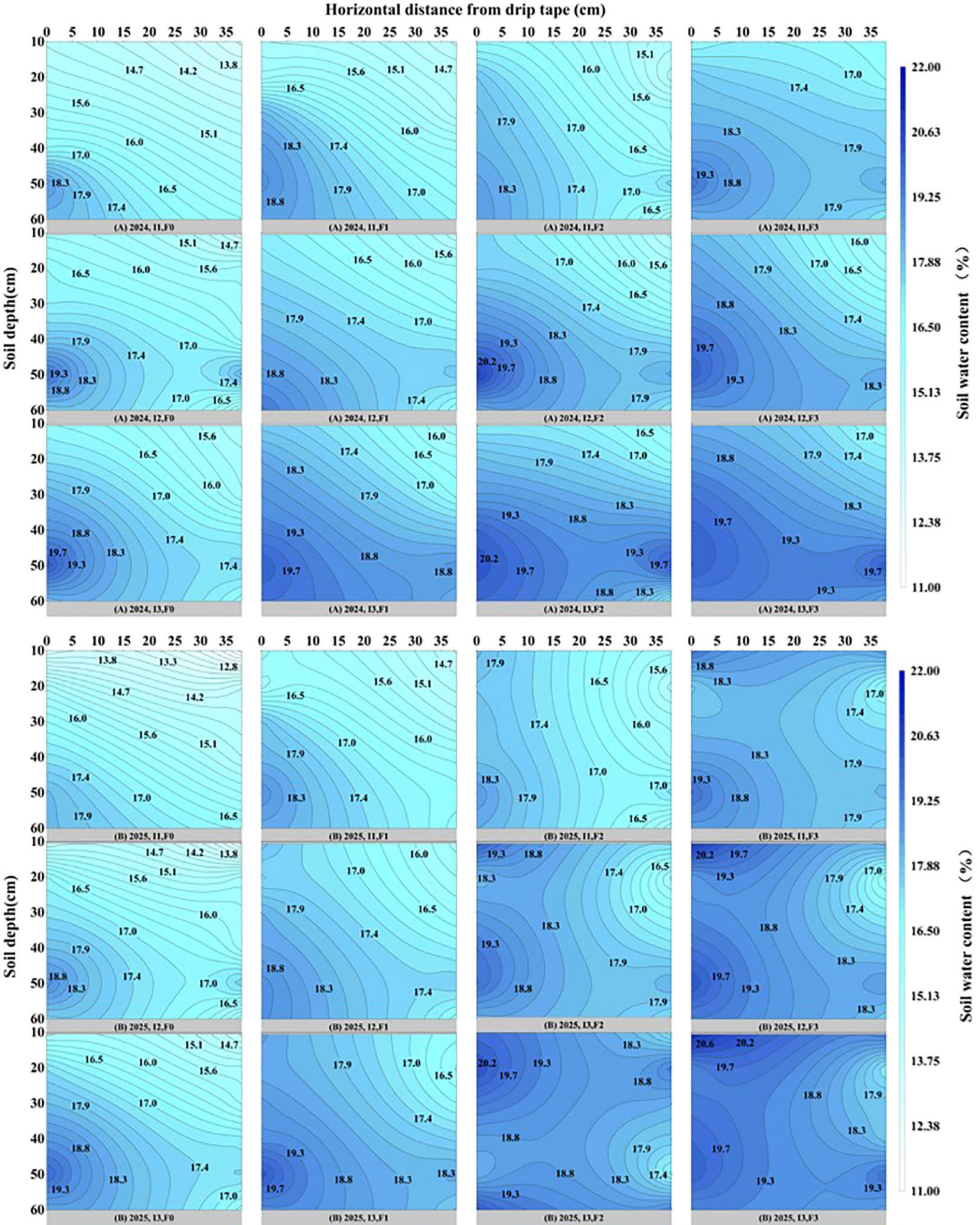
Water distribution in cotton fields contaminated with residual film under different irrigation levels and C/N ratios in 2024-2025.The mean irrigation amounts of I1, I2, and I3 correspond to 0.6 ET_c_, 0.8 ET_c_, and 1.0 ET_c_, respectively. The C/N ratios of F0, F1, F2, and F3 are 0:1, 3:1, 6:1, and 9:1. **(A)** and **(B)** represent the data for 2024 and 2025, respectively.

Further analysis of the spatial distribution of SWC (**Figure 3**) revealed that increasing the C/N ratio significantly increased the soil water content in the plow layer of cotton fields contaminated with residual film. Horizontally, the average SWC directly beneath the dripper was 1.2%-21.0% higher than at a position 38 cm laterally from the dripper. Vertically, increasing the C/N application ratio significantly increased SWC in the upper and middle layers (0–40 cm). Under I1 conditions, F3 showed a 6.6%-19.9% increase in SWC in the upper and middle layers compared to other treatments. under I2 conditions, F3 increased SWC in the upper and middle layers by 13.4% compared to F0; under I3 conditions, F2 increased SWC in the upper and middle layers by 9.6% compared to F0, which was only 2.5% lower than F3. Under the interaction of irrigation volume and C/N ratio, the SWC of I2F2 increased by 4.8% at a lateral distance of 38 cm from the dripper compared to I3F0 in the horizontal direction, and the SWC of the upper and middle soil layers increased by 5.3% compared to I3F0 in the vertical direction. Therefore, increasing the C/N ratio promotes water movement in both the horizontal and vertical directions within the soil of cotton fields contaminated by plastic film residues.

### 3.2 Soil water content uniformity coefficient

Both the C/N ratio and irrigation rate had a significant effect (p< 0.05) on the soil water content uniformity coefficient (C*u*) in cotton fields contaminated with plastic film residues (**Table 3**). The two-year study indicated that increasing the C/N application ratio can improve the soil water content uniformity coefficient in cotton fields contaminated with plastic film residues. Under condition I1, the increase in C*u* (SWC) for F3 was 1.8%-14.1% higher than that of other C/N treatments; under condition I2, F2 was 9.5% higher than F0 but 1.3% lower than F3; and under condition I3, F2’s C*u* was 0.94, which was 9.1% higher than F0 and only 0.1% lower than F3. Under the combined effects of irrigation volume and C/N ratio, the C*u* (SWC) in the 0–60 cm soil layer of I2F2 was 5.4% lower than that of I3F3, but 4.2% higher than that of I3F0. Specifically, the C*u* (SWC) in the 0–40 cm layer of I2F2 was 0.88, which was 4.7% higher than that of I3F0. The C/N ratio and irrigation volume significantly increased the coefficient of uniformity (C*u*) of soil water content (SWC) in cotton fields contaminated by residual plastic film.

**Table 3:** Coefficient of uniformity of water distribution in cotton fields contaminated with residual film under different irrigation levels and C/N ratios.

Year	Irrigation	Fertilization	Soil depth (cm)						
			0-10	10-20	20-30	30-40	40-50	50-60	0-60
2024	I1	F0	0.69 h	0.74 h	0.78 e	0.87 i	0.84 gh	0.85 e	0.79 h
		F1	0.71 gh	0.78 g	0.81 de	0.89 gh	0.84 gh	0.85 e	0.81 g
		F2	0.76 f	0.82 ef	0.88 bc	0.90 fg	0.85 fg	0.91 cd	0.85 e
		F3	0.79 ef	0.85 bcd	0.89 bc	0.91 ef	0.87 de	0.91 d	0.87 d
	I2	F0	0.73 g	0.80 fg	0.85 cd	0.88 hi	0.83 h	0.91 d	0.83 f
		F1	0.80 e	0.84 cde	0.88 bc	0.89 g	0.86 ef	0.93 bcd	0.87 de
		F2	0.83 cd	0.87 bc	0.88 bc	0.92 de	0.86 de	0.95 ab	0.89 c
		F3	0.85 bc	0.88 b	0.89 bc	0.93 cd	0.90 c	0.94 abc	0.90 c
	I3	F0	0.81 de	0.84 de	0.85 c	0.90 fg	0.87 d	0.92 cd	0.87 d
		F1	0.86 b	0.91 a	0.92 ab	0.94 bc	0.91 b	0.96 a	0.92 b
		F2	0.91 a	0.93 a	0.94 a	0.95 ab	0.94 a	0.93 bcd	0.93 a
		F3	0.92 a	0.93 a	0.95 a	0.96 a	0.94 a	0.97 a	0.95 a
2025	I1	F0	0.48 f	0.75 f	0.80 d	0.85 e	0.82 g	0.83 f	0.75 j
		F1	0.76 cd	0.77 f	0.81 cd	0.89 cd	0.84 f	0.86 e	0.82 h
		F2	0.90 ab	0.80 e	0.85 bc	0.92 bc	0.88 e	0.94 cd	0.88 f
		F3	0.95 a	0.81 de	0.86 b	0.93 ab	0.89 d	0.93 cd	0.90 e
	I2	F0	0.58 e	0.81 de	0.86 b	0.86 e	0.83 fg	0.89 e	0.81 i
		F1	0.84 bc	0.83 cd	0.87 b	0.90 cd	0.87 e	0.93 d	0.87 fg
		F2	0.92 ab	0.84 bcd	0.88 b	0.93 ab	0.90 d	0.98 a	0.91 de
		F3	0.94 a	0.85 bc	0.88 b	0.95 a	0.93 c	0.97 ab	0.92 cd
	I3	F0	0.72 d	0.86 b	0.88 b	0.89 d	0.87 e	0.92 d	0.86 g
		F1	0.91 ab	0.90 a	0.92 a	0.95 a	0.93 c	0.97 abc	0.93 bc
		F2	0.96 a	0.93 a	0.95 a	0.94 a	0.95 b	0.94 bcd	0.94 ab
		F3	0.96 a	0.93 a	0.94 a	0.94 a	0.97 a	0.97 a	0.95 a
Source of variance									
Year (Y)			**	NS	NS	NS	**	NS	**
Irrigation amounts (I)			**	**	**	**	**	**	**
Fertilization treatment (F)			**	**	**	**	**	**	**
I×F			NS	NS	*	*	**	**	**
Y×I×F			NS	NS	NS	NS	NS	NS	NS

For a given trait, treatments with the same lowercase letters within a year were not significantly different based on Duncan’s multiple range test at p< 0.05 with a general linear model. * and ** represent a significant difference at the 5 and 1% levels, respectively; NS represents no significant difference at the 5% level. The mean irrigation amounts of I1, I2, and I3 correspond to 0.6 ET_c_, 0.8 ET_c_, and 1.0 ET_c_, respectively. The C/N ratios of F0, F1, F2, and F3 are 0:1, 3:1, 6:1, and 9:1, respectively.

### 3.3 Soil salinity content and distribution

**Figure 4:**
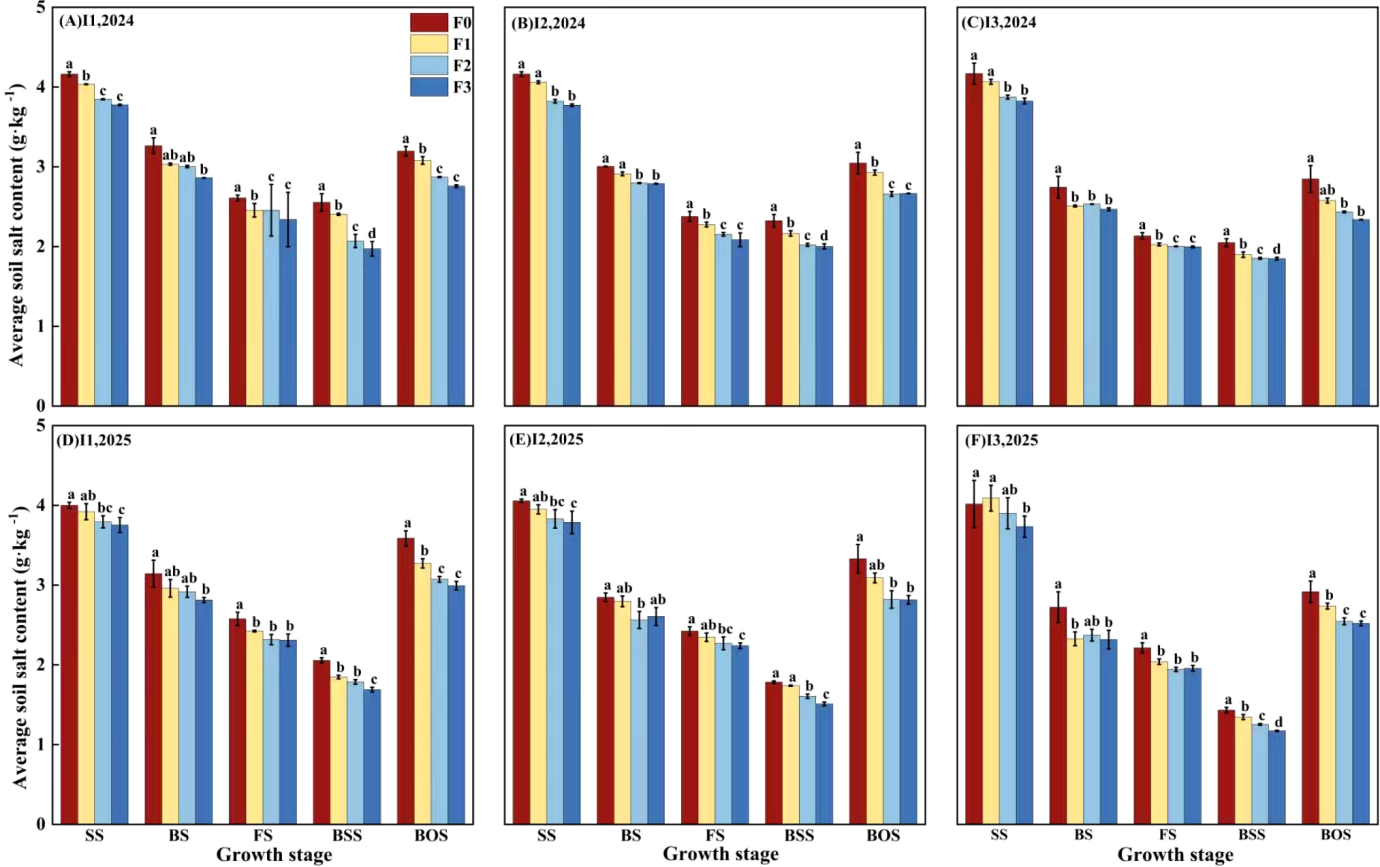
Average soil salinity (0–60 cm) in cotton fields contaminated by pesticide residues under different irrigation levels and C/N ratios. The error bars represent the standard error of three replicates. Values with different lowercase letters are significantly different at P<0.05 using the LSD method. The average irrigation levels for I1, I2, and I3 were 0.6 ET_c_, 0.8 ET_c_, and 1.0 ET_c_, respectively. The C/N ratios for F0, F1, F2, and F3 were 0:1, 3:1, 6:1, and 9:1, respectively. SS denotes the seedling stage, BS denotes the budding stage, FS denotes the flowering stage, BSS denotes the boll stage, and BOS denotes the boll opening stage. **(A–C)** and **(D–F)** represent different irrigation treatments (I1, I2, and I3) in 2024 and 2025, respectively.

Increasing the C/N ratio can significantly affect soil salinity content (SSC) in cotton fields contaminated by plastic film residues (**Figure 4**). Two years of research indicate that under condition I1, the SSC in treatment F3 was 3.2%-12.7% lower than in other C/N treatments; under condition I2, the SSC in treatment F2 was 9.9% lower than in F0 and only 1.2% higher than in F3; and under condition I3, the SSC in treatment F3 was 3.7%-14.3% lower than in other C/N treatments. Under the combined effects of C/N ratio and irrigation volume, the SSC of I2F2 was 1.9% lower than that of I3F0, but 2.2% higher than that of I3F3. Increasing the C/N ratio can significantly reduce the soil salinity content (SSC) in cotton fields contaminated by residual plastic film.

**Figure 5:**
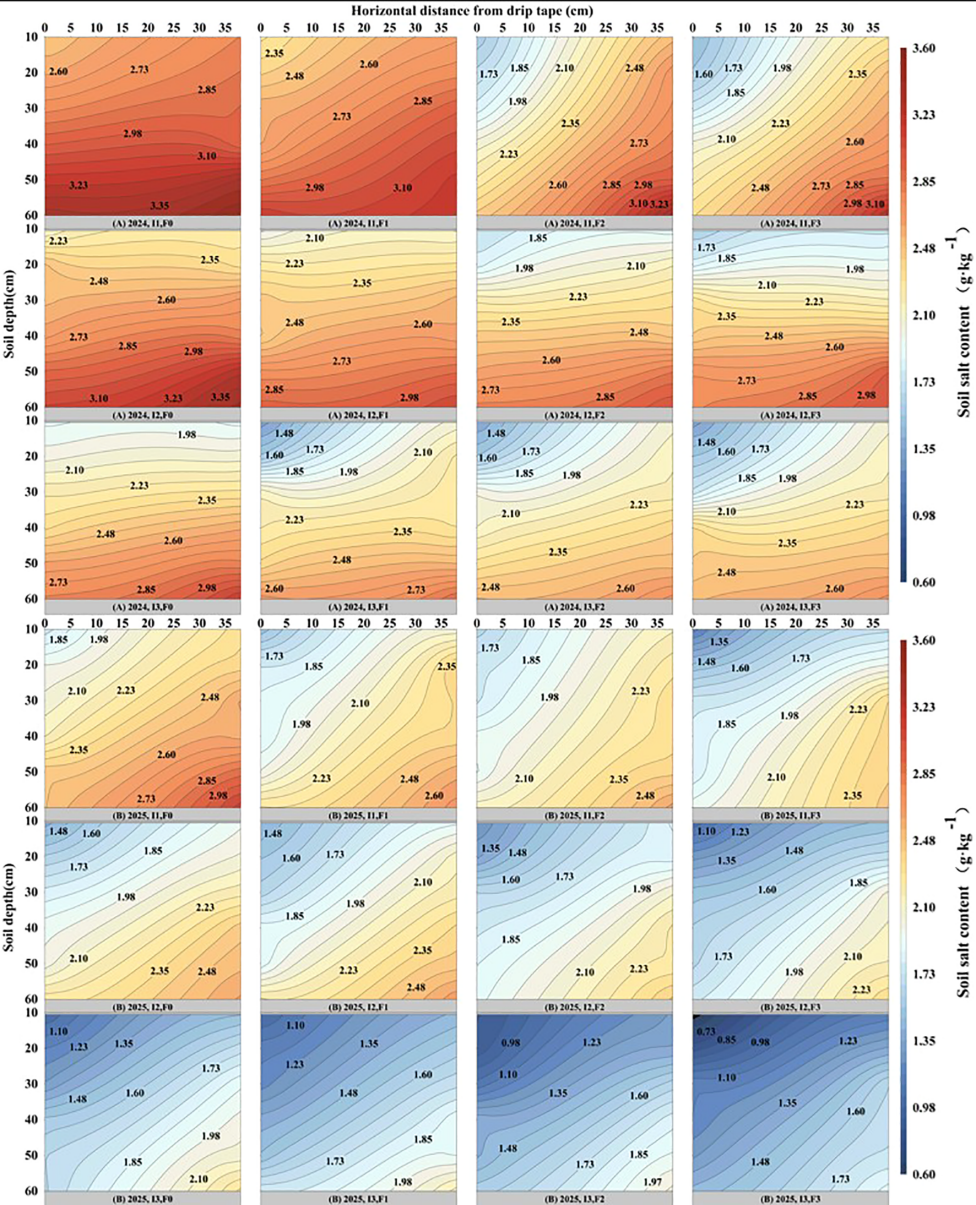
Salt distribution (0–60 cm) in cotton fields contaminated by residual film under different irrigation levels and C/N ratios (2024-2025).The mean irrigation amounts of I1, I2, and I3 correspond to 0.6 ET_c_, 0.8 ET_c_, and 1.0 ET_c_, respectively. The C/N ratios of F0, F1, F2, and F3 are 0:1, 3:1, 6:1, and 9:1. **(A)** and **(B)** represent the data for 2024 and 2025, respectively.

Further analysis of the spatial distribution of SSC revealed (**Figure 5**) that, in the horizontal direction, the average SSC directly beneath the dripper was 10.9%-43.1% lower than at a point 38 cm laterally from the dripper. Vertically, increasing the C/N application ratio significantly reduced SSC in the upper and middle layers (0–40 cm). Under I1 conditions, SSC in the upper and middle layers of F3 was 8.2%-20.6% lower than in other treatments. under I2 conditions, SSC in the upper 0–40 cm layer of F2 was 11.7% lower than that of F0 and only 6.2% higher than that of F3; under I3 conditions, SSC in the upper 0–40 cm layer of F3 was 4.6%-16.0% lower than that of other treatments. Under the interaction of irrigation volume and C/N ratio, the average SSC in the 0–40 cm soil layer for I2F2 was 1.86 g kg^-^¹, accounting for 61.3% of the SSC in the 0–60 cm soil layer. In summary, increasing the C/N ratio promotes the transport of soil salts in cotton fields contaminated by plastic film residues, significantly reducing salt content in the upper and middle soil layers.

### 3.4 Coefficient of uniformity of soil salinity

Both the C/N ratio and irrigation rate had a significant effect (p< 0.05) on the SSC uniformity coefficient (C*u*) in cotton fields contaminated with plastic film residues (**Table 4**). This study indicates that increasing the C/N ratio significantly improves the SSC uniformity coefficient (C*u*) in the 0–60 cm soil layer. Under I1 conditions, the C*u* (SSC) of F3 was 2.0%-7.3% higher than that of other C/N treatments; under I2 conditions, F2’s C*u* (SSC) was 4.7% higher than F0’s and only 2.8% lower than F3’s; while under I3 conditions, F3’s C*u* (SSC) was 3.5%-8.9% higher than that of other C/N treatments. Additionally, under the same irrigation rate, in the 0–40 cm soil layer, C*u* (SSC) in F3 increased by 3.8%-10.9% compared to other C/N ratios. Under the combined regulation of C/N ratio and irrigation volume, the C*u* (SSC) of I2F2 in the 0–40 cm soil layer reached 0.84, which was only 1.3% lower than that of I3F0. The C/N ratio can promote salt transport in the soil, significantly improving the uniformity of soil salinity in cotton fields contaminated by residual plastic film.

**Table 4:** Coefficient of uniformity of salt distribution in cotton fields contaminated by residual film under different irrigation levels and C/N ratios.

Year	Irrigation	Fertilization	Soil depth (cm)						
			0-10	10-20	20-30	30-40	40-50	50-60	0-60
2024	I1	F0	0.71 ef	0.75 ef	0.75 e	0.76 f	0.82 f	0.81 f	0.76 g
		F1	0.66 f	0.69 f	0.80 d	0.81 e	0.84 e	0.91 ab	0.78 fg
		F2	0.81 bcd	0.83 cd	0.82 cd	0.83 de	0.86 e	0.69 g	0.81 ef
		F3	0.79 cd	0.83 cd	0.82 cd	0.82 de	0.86 e	0.82 ef	0.83 de
	I2	F0	0.75 de	0.71 f	0.81 cd	0.83 de	0.86 e	0.89 abc	0.81 ef
		F1	0.81 cd	0.80 de	0.81 cd	0.83 de	0.87 de	0.84 def	0.83 de
		F2	0.80 cd	0.85 bcd	0.85 bc	0.86 cd	0.89 cd	0.87 cde	0.85 cd
		F3	0.83 bc	0.91 ab	0.87 b	0.88 c	0.90 c	0.86 cde	0.87 bc
	I3	F0	0.84 bc	0.85 bcd	0.84 bc	0.86 cd	0.89 c	0.85 def	0.85 cd
		F1	0.88 b	0.91 ab	0.86 b	0.86 cd	0.90 c	0.86 cde	0.88 bc
		F2	0.81 bcd	0.87 bc	0.81 cd	0.99 a	0.99 a	0.95 a	0.90 ab
		F3	0.96 a	0.97 a	0.92 a	0.93 b	0.95 b	0.88 bcd	0.93 a
2025	I1	F0	0.70 ef	0.75 fg	0.74 g	0.76 f	0.84 gh	0.80 e	0.77 g
		F1	0.65 f	0.68 h	0.78 f	0.80 ef	0.84 gh	0.94 ab	0.78 fg
		F2	0.81 bcd	0.83 de	0.82 def	0.83 de	0.86 fg	0.67 f	0.81 efg
		F3	0.80 cd	0.82 def	0.80 ef	0.83 de	0.86 fg	0.80 e	0.82 ef
	I2	F0	0.74 de	0.72 gh	0.83 cde	0.83 de	0.83 h	0.91 abc	0.81 efg
		F1	0.81 bcd	0.78 efg	0.82 def	0.83 de	0.88 ef	0.85 cde	0.83 de
		F2	0.78 cde	0.85 cde	0.86 bc	0.87 cd	0.89 ef	0.83 de	0.84 cde
		F3	0.83 bc	0.91 abc	0.88 b	0.85 cd	0.90 de	0.85 cde	0.87 bcd
	I3	F0	0.84 bc	0.85 cde	0.84 bcd	0.88 c	0.92 cd	0.83 de	0.86 bcd
		F1	0.88 b	0.92 ab	0.86 bc	0.85 cd	0.93 bc	0.85 cde	0.88 bc
		F2	0.82 bc	0.88 abc	0.80 ef	0.97 a	0.98 a	0.96 a	0.90 ab
		F3	0.97 a	0.95 a	0.92 a	0.93 b	0.95 ab	0.88 bcd	0.93 a
Source of variance									
Year(Y)			**	NS	**	NS	NS	**	**
Irrigation amounts(I)			**	**	**	**	**	**	**
Fertilization treatment(F)			**	**	**	**	**	*	**
I×F			**	**	**	NS	NS	**	*
Y×I×F			NS	NS	NS	NS	NS	**	*

For a given trait, treatments with the same lowercase letters within a year were not significantly different based on Duncan’s multiple range test at p< 0.05 with a general linear model. * and ** represent a significant difference at the 5 and 1% levels, respectively; Ns represents no significant difference at the 5% level. The mean irrigation amounts of I1, I2, and I3 correspond to 0.6 ET_c_, 0.8 ET_c_, and 1.0 ET_c_, respectively. The C/N ratios of F0, F1, F2, and F3 are 0:1, 3:1, 6:1, and 9:1, respectively.

### 3.5 Desalination rate

Both irrigation rate and the C/N ratio significantly affected the desalination rate (Rs) in the 0–60 cm soil layer (p< 0.05) (**Table 5**). This study indicates that under condition I1, the Rs of treatment F3 was on average 66.7% higher than that of treatment F0. Under condition I2, treatment F2 had the highest Rs, reaching 32.6%, which was 3.9%-37.4% higher than that of other C/N treatments; under condition I3, the Rs of treatment F2 was 5.6%-23.2% higher than that of other C/N treatments. Additionally, under the same irrigation rate, in the 0–40 cm soil layer, there was no significant difference in Rs between the F3 and F2 treatments, which were 7.6%-50.8% lower than other C/N treatments. Under the interaction of C/N ratio and irrigation volume, the Rs of I2F2 was 16.5% lower than that of I3F3, showed no significant difference from I3F0, and was 4.0% higher than I2F3; among these, the average Rs of I2F2 in the 0–40 cm soil layer reached 0.485, an increase of 0.5% compared to I3F0. Therefore, an appropriate C/N ratio significantly promoted soil salt leaching.

**Table 5:** Desalination rates (%) in different soil layers of cotton fields contaminated with residual film under varying irrigation levels and C/N ratios.

Year	Treatment		Soil depth (cm)						
			0-10	10-20	20-30	30-40	40-50	50-60	0-60
2024	I1	F0	0.43 h	0.41 e	0.20 a	0.20 h	-0.14 f	-0.08 c	0.17 g
		F1	0.55 f	0.52 c	0.27 ab	0.24 gh	-0.12 ef	-0.16 d	0.22 fg
		F2	0.60 de	0.56 b	0.30 bc	0.26 efg	-0.11 def	-0.12 cd	0.25 ef
		F3	0.62 cde	0.56 bc	0.31 bcd	0.32 cde	-0.06 cde	-0.16 d	0.26 def
	I2	F0	0.49 g	0.45 d	0.28 cde	0.25 fgh	-0.07 cde	0.02 b	0.24 f
		F1	0.59 e	0.56 b	0.36 cde	0.30 def	-0.06 cde	0.00 b	0.29 cde
		F2	0.63 cd	0.59 b	0.36 def	0.36 bc	-0.02 abc	0.03 ab	0.32 c
		F3	0.64 c	0.58 b	0.35 efg	0.34 cd	-0.04 bcd	-0.01 b	0.31 cd
	I3	F0	0.63 cde	0.58 b	0.39 fg	0.33 cd	0.01 abc	0.09 a	0.34 bc
		F1	0.71 b	0.68 a	0.42 g	0.42 a	0.04 a	0.08 a	0.39 a
		F2	0.77 a	0.71 a	0.47 g	0.42 ab	0.01 ab	0.05 ab	0.40 a
		F3	0.75 ab	0.68 a	0.40 h	0.45 a	0.01 ab	0.02 ab	0.39 ab
2025	I1	F0	0.41 f	0.40 e	0.21 g	0.18 g	-0.18 d	-0.12 cde	0.15 f
		F1	0.56 d	0.52 cd	0.30 ef	0.24 fg	-0.12 cd	-0.16 de	0.22 e
		F2	0.60 cd	0.55 bc	0.27 fg	0.28 def	-0.09 bcd	-0.09 cde	0.26 de
		F3	0.63 c	0.56 bc	0.32 def	0.36 bcd	-0.10 bcd	-0.16 e	0.27 cde
	I2	F0	0.50 e	0.47 d	0.30 ef	0.25 fg	-0.10 bcd	-0.03 abc	0.23 de
		F1	0.61 cd	0.55 bc	0.36 cde	0.27 ef	-0.09 bcd	-0.02 abc	0.28 bcd
		F2	0.63 c	0.60 b	0.35 def	0.36 bc	0.01 ab	0.01 abc	0.33 bc
		F3	0.64 c	0.59 b	0.33 def	0.31 cde	-0.02 abc	0.05 a	0.32 bc
	I3	F0	0.65 c	0.56 bc	0.39 bcd	0.32 cde	0.00 ab	0.06 a	0.33 b
		F1	0.71 b	0.68 a	0.41 abc	0.43 ab	0.06 a	0.09 a	0.39 a
		F2	0.78 a	0.72 a	0.48 a	0.45 a	0.04 a	0.04 ab	0.42 a
		F3	0.75 ab	0.67 a	0.44 ab	0.42 ab	0.05 a	0.04 ab	0.39 a
Source of variance									
Year (Y)			**	**	**	NS	**	**	**
Irrigation amounts (I)			**	**	**	**	**	**	**
fertilization treatment (F)			**	**	**	**	**	**	**
I×F			**	NS	**	**	NS	*	*
Y×I×F			NS	NS	NS	NS	NS	NS	NS

For a given trait, treatments with the same lowercase letters within a year were not significantly different based on Duncan’s multiple range test at p< 0.05 with a general linear model. * and ** represent a significant difference at the 5 and 1% levels, respectively; Ns represents no significant difference at the 5% level. The mean irrigation amounts of I1, I2, and I3 correspond to 0.6 ET_c_, 0.8 ET_c_, and 1.0 ET_c_, respectively. The C/N ratios of F0, F1, F2, and F3 are 0:1, 3:1, 6:1, and 9:1, respectively.

### 3.6 Dry matter accumulation, seed cotton yield and harvest index

**Figure 6:**
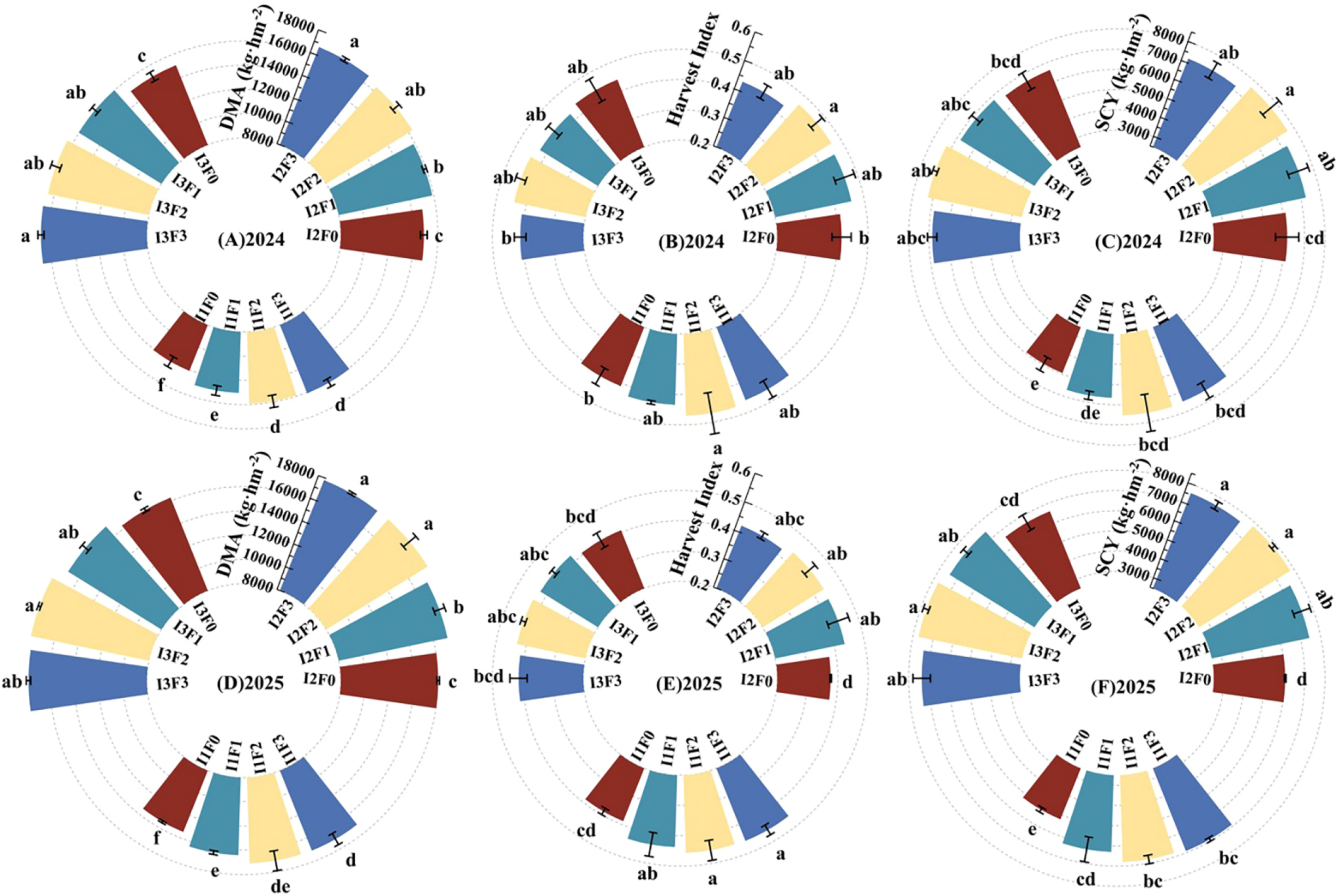
Total dry matter accumulation (kg·hm^-2^), harvest index, and seed cotton yield (kg·hm^-2^) in cotton fields contaminated with residual film under different irrigation levels and C/N ratios. DMA, harvest index, and SCY refer to total dry matter accumulation, harvest index, and seed cotton yield. The error bars represent the standard error of three replicates. Values with different lowercase letters are significantly different at P<0.05 using the LSD method. The mean irrigation amounts of I1, I2, and I3 correspond to 0.6 ET_c_, 0.8 ET_c_, and 1.0 ET_c_, respectively. The C/N ratios of F0, F1, F2, and F3 are 0:1, 3:1, 6:1, and 9:1.

Increasing the C/N ratio promoted the accumulation and distribution of dry matter in cotton fields contaminated with plastic film residues, thereby enhancing cotton yield (**Figure 6**). Under the same irrigation conditions, the dry matter accumulation (DMA) of F3 increased by 0.01%-29.2% compared to other treatments, but the harvest index (HI) decreased by 2.0%-5.5% compared to F2, resulting in lower seed cotton yield (SCY). Specifically, under I1 conditions, the SCY of F2 increased by 0.4%-31.9% compared to other C/N treatments; Under I2 conditions, F2’s SCY was 27.0% higher than F0’s; under I3 conditions, F2’s SCY was 13.3% higher than F0’s. Under the interaction of irrigation volume and C/N ratio, the DMA of I2F2 increased by an average of 0.8% compared to I3F3, and the HI of I3F3 and I3F0 decreased by 6.6% and 9.9%, respectively, compared to I2F2, resulting in an average increase in SCY for I2F2 of 9.0% and 17.9% compared to I3F3 and I3F0, respectively. In summary, an appropriate C/N ratio can promote yield formation.

### 3.7 Relationship between soil water and salinity characteristics and seed cotton yield

**Figure 7:**
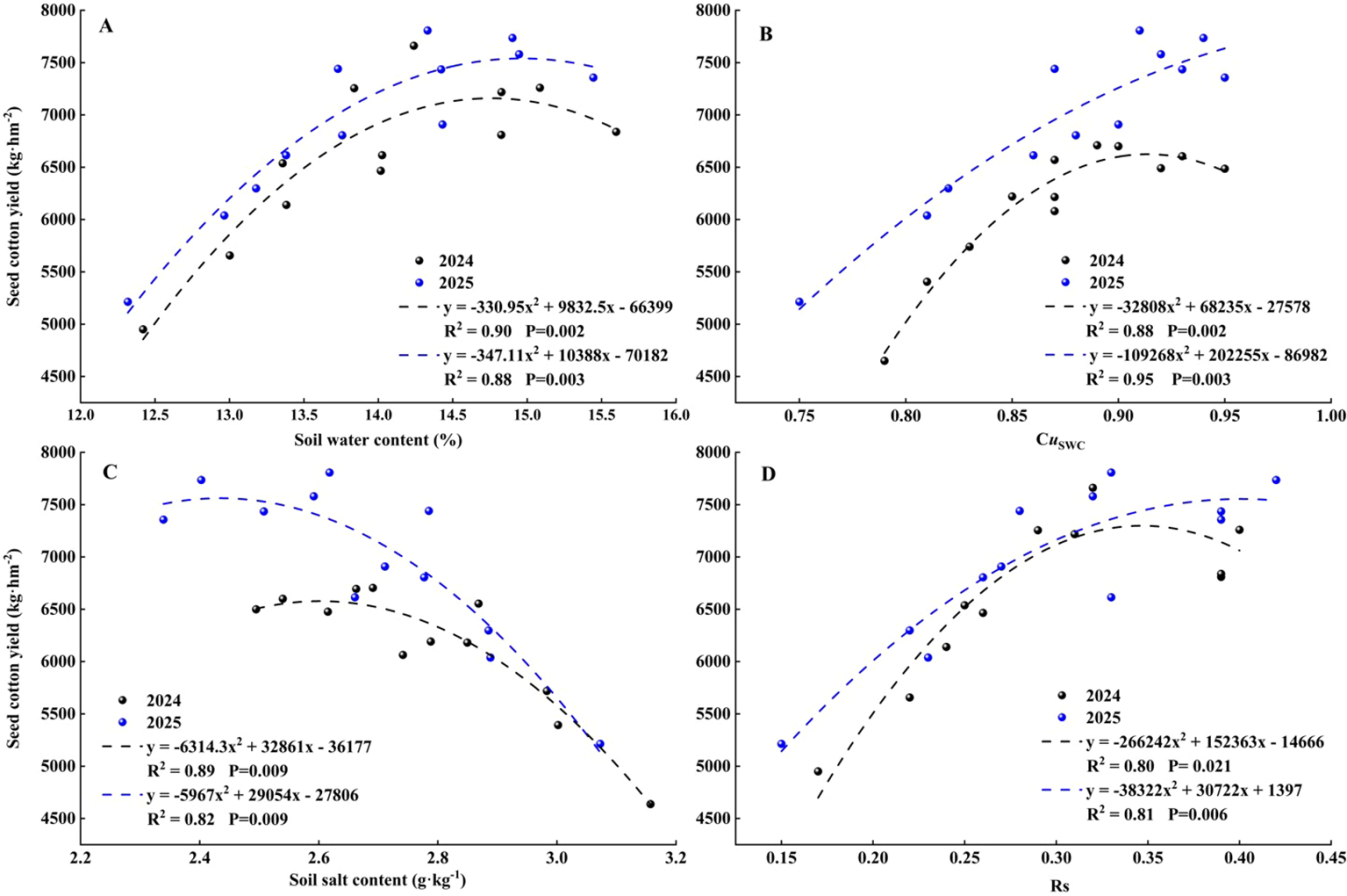
Correlation analysis between soil water and salinity characteristics and seed cotton yield under different irrigation levels and C/N ratios in cotton fields contaminated with pesticide residues. **(A)** soil water content, **(B)** Cuswc, **(C)** soil salt content, and **(D)** Rs.

Regression analysis indicated that seed cotton yield exhibited a significant quadratic relationship with soil water content (SWC), the coefficient of uniformity of soil water content (C*u*_SWC_), soil salinity (SSC), and the desalination rate (Rs) (p< 0.05) (**Figure 7**). In 2024–2025, the maximum seed cotton yield was 7085.21 kg·hm^-2^, achieved at an SWC of 14.87%.(**Figure 7A**); when C*u*_SWC_ was 0.96, yield peaked at 7256.37 kg·hm^-2^ (**Figure 7B**); and when Rs was 0.36, yield was highest at 7343.30 kg·hm^-2^ (**Figure 7D**). Further increases in SWC, C*u*_SWC_, or Rs did not significantly increase seed cotton yield and even showed a declining trend. For SSC, seed cotton yield was highest at 7068.89 kg·hm^-2^ when SSC was 2.5 g kg^-^¹ (**Figure 7C**); further reductions in SSC did not significantly increase yield. Overall, the relationships between SWC, C*u*_SWC_, and Rs and seed cotton yield all exhibited a pattern of rapid initial increase followed by a decline in the rate of increase, or even a downward trend, whereas the relationship between SSC and seed cotton yield showed a negative correlation.

## 4 Discussion

Under conditions of cotton fields contaminated by residual plastic film, at the same irrigation rate, a C/N ratio of 6:1 resulted in a 3.4%–23.1% increase in seed cotton yield compared to other ratios. At the same C/N ratio, as the irrigation rate increased, soil moisture content and its uniformity, soil salinity uniformity, desalination rate, and seed cotton yield all showed an upward trend. There was a significant interaction between irrigation volume and C/N ratio, with the “slightly deficient irrigation + C/N ratio of 6:1” combination yielding the highest seed cotton yield, 1.2% higher than that of “full irrigation + C/N ratio of 9:1.” These results indicate that in cotton fields contaminated with residual plastic film, moderately reducing irrigation and optimizing the C/N ratio can maintain high yields while conserving water.

### 4.1 Effects of water-carbon interactions on soil moisture in cotton fields contaminated by residual film

In arid and water-scarce regions, optimizing irrigation regimes and improving soil structure are key strategies for enhancing crop water use efficiency. Furthermore, replacing chemical fertilizers with organic fertilizers and adjusting the C/N ratio play a crucial role in alleviating water shortages and improving soil water-holding capacity (Xu et al., 2023).This study indicates that as irrigation volume increases, soil moisture content rises significantly, the depth of the effective moisture zone expands (**Figure 3**), and the spatiotemporal uniformity of moisture (C*u*) improves markedly (**Table 2**). This may be because increased irrigation volume allows the moisture front to advance further (Faloye et al., 2025), thereby expanding the water-available zone for the root system. Concurrently, post-irrigation water redistribution driven by capillary action and gravity reduces vertical and horizontal variations in soil moisture (Li et al., 2010).Furthermore, this study found that under identical irrigation conditions, increasing the C/N ratio in fertilizer application also significantly improved soil moisture content and C*u*. This aligns with the findings of most studies on organic fertilizer amendment (Wei et al., 2023), as a higher C/N ratio significantly increases soil organic matter content, improves aggregate structure and pore distribution, enhances soil capillarity, thereby enhancing field water-holding capacity and water retention ability, and making water distribution more uniform both vertically and horizontally (Hao et al., 2020). Meanwhile, an appropriate C/N ratio can promote microbial growth, enhance organic matter decomposition and nutrient mineralization, optimize C and N cycling, and improve soil nutrient supply and ecological functions (Chen et al., 2025b). Under the interaction of these two factors, our results showed that, notably, under mild water-deficient irrigation conditions, soil moisture levels in the 6:1 C/N treatment (F2) showed no significant difference compared to those in the fully irrigated + 0:1 C/N treatment (**Figure 2**), indicating that an appropriate organic fertilizer ratio can partially compensate for insufficient irrigation water (Liu et al., 2025). This may be because the combined effects of increased C/N ratios on improving soil structure and water-holding capacity are more readily evident under water-deficient conditions (Alves et al., 2024).However, when the C/N ratio was further increased to 9:1 (F3), there were no significant differences in soil moisture content and C*u* levels compared to the 6:1 (F2) treatment. This finding is inconsistent with some long-term organic fertilizer trial results (Zhang et al., 2022a) which may be because the improvement in soil physical properties resulting from high C/N ratios requires long-term accumulation, and this process may be significantly delayed under plastic film residue contamination (Qi et al., 2020). These conclusions were reached through a two-year experimental study; however, to investigate the long-term regulatory effects of deficit irrigation and different carbon-to-nitrogen ratios on water and salt distribution under conditions of residual film contamination, further research could be conducted through long-term fixed-site and multi-site experiments.

### 4.2 Effects of water-carbon interactions on soil salinity in cotton fields contaminated by residual pesticide films

Soil moisture is closely related to salt transport processes (Corwin and Lesch, 2005).In arid and water-scarce regions, low irrigation levels can lead to surface salt accumulation due to evaporation, exacerbating osmotic stress and ion toxicity; conversely, moderate or high irrigation can increase soil moisture content and deepen the penetration of the moisture front (Ortiz and Jin, 2021), promoting the leaching of deep-seated salts and reducing surface salt accumulation, thereby improving the salinity environment in the root zone (Yang et al., 2023).This study further confirmed under conditions of cotton fields contaminated by plastic film residues that increasing irrigation levels can effectively reduce soil salinity while improving the uniformity of salt distribution and desalination rate (**Tables 4**, **5**). Meanwhile, the application of organic fertilizer can enhance the soil’s buffering capacity against salts, thereby slowing the accumulation of salts in the arable layer (Liu et al., 2023b). This is because an appropriate C/N ratio can improve soil organic matter content and aggregate structure, thereby enhancing the soil’s buffering and regulatory capacity against salinity (Song et al., 2025). This helps reduce excessive salt accumulation in the soil, promotes uniform salt distribution, and leads to more efficient desalination. The results of this study also support this view, indicating that increasing the C/N ratio in cotton fields contaminated with plastic film residues has a positive effect on soil desalination. Further analysis of the interactive effects between irrigation volume and C/N application ratio revealed that the spatial uniformity of salt distribution and desalination rate for the “slightly deficit irrigation + C/N ratio of 6:1” combination were essentially consistent with those of the “full irrigation + C/N ratio of 0:1” combination, This indicates that in cotton fields contaminated by plastic film residues, appropriately adjusting the C/N ratio of fertilizers can enhance water-salt regulation capacity under deficit irrigation conditions, thereby achieving the dual benefits of water conservation and salt control. Through two years of experiments, this study has determined the effects of irrigation volume and C/N ratio regulation on water and salt transport in cotton fields contaminated with plastic film residues; however, the impact on other physicochemical properties remains unclear. Future research could further integrate long-term field experiments to systematically evaluate the sustained effects of different irrigation levels and C/N regulation on soil bulk density, porosity, aggregate stability, organic matter, nutrient availability, and salt leaching processes in cotton fields contaminated by plastic film residues, thereby elucidating the long-term mechanisms underlying their impact on soil quality and sustainable productivity in cotton fields.

### 4.3 Effects of water-carbon interactions on yield in cotton fields contaminated by residual pesticide films

Soil moisture and salinity conditions jointly determine cotton growth, development, and yield formation (Ren et al., 2021).Water deficiency reduces photosynthetic efficiency and nutrient uptake, inhibiting dry matter accumulation and its allocation to reproductive organs (Chen et al., 2024);excessive soil salinity, on the other hand, impairs germination and growth through osmotic stress and ionic toxicity, thereby reducing both yield and quality (Isayenkov and Maathuis, 2019).In arid and water-scarce regions, appropriate irrigation levels can both mitigate salt damage and promote yield formation, serving as a core measure for achieving coordinated water-salt regulation and ensuring stable, high yields (Yu et al., 2020). These findings are largely consistent with the results of this study under identical fertilization conditions. At the same time, the slow-release properties of organic fertilizers can provide nutrients continuously throughout the crop growth cycle, compensating for the “nutrient depletion” phenomenon observed with chemical fertilizers in the later stages, thereby benefiting yield formation (Xu et al., 2026);In this study, conducted in cotton fields contaminated with plastic film residues and under identical irrigation conditions, the seed cotton yield with a C/N ratio of 6:1 was significantly higher than that of other fertilization treatments. This may be because a low C/N ratio limits organic matter accumulation, while a high C/N ratio leads to N fixation and reduced available N supply, thereby inhibiting yield increases (Liu et al., 2022). Fertilization with an appropriate carbon-to-nitrogen (C/N) ratio can effectively improve the accumulation of photosynthetic products in cotton and, by increasing the utilization efficiency of nitrogen, promote the transfer and allocation of dry matter to reproductive organs (Abudurezike et al., 2025). Particularly under conditions of moderate water stress, a reasonable C/N ratio helps mitigate nitrogen fixation and leaching, maintains a stable supply of nitrogen, and consequently enhances cotton growth vigor and boll-setting rates, thereby promoting yield formation. Furthermore, an appropriate nitrogen supply not only improves crop growth but also optimizes the soil environment and reduces greenhouse gas emissions (Chen et al., 2025a).Under the combined regulation of irrigation volume and fertilizer ratios, this further demonstrates that water-fertilizer synergy not only improves water and salt distribution in the root zone but also promotes dry matter accumulation and its effective allocation to reproductive organs, thereby facilitating crop yield formation. Furthermore, analysis of lint yield and soil water-salt characteristics revealed that regulating soil moisture can effectively mitigate salt damage and increase cotton yield. Therefore, by optimizing irrigation regimes and organic fertilizer ratios, combined with water-salt synergistic regulation, cotton yields can be significantly enhanced in special environments such as fields contaminated by residual plastic film, providing practical management strategies for cotton production in arid and semi-arid regions. Based on two years of experimental data, this study preliminarily demonstrates that optimizing irrigation regimes and combining carbon and nitrogen application with coordinated water-salt regulation can stabilize and increase cotton yields in fields contaminated with plastic film residues; however, the underlying mechanisms—such as microbial activity and enzyme activity in the soil environment, as well as nutrient uptake and utilization by plants—require further investigation. Future research could utilize long-term field trials to further validate the long-term effects of different irrigation and fertilization regimes under varying climatic and soil conditions. By thoroughly investigating the combined effects of dynamic changes in water-salt and nutrient management on cotton growth and yield, more precise management strategies can be developed to support efficient and sustainable cotton production in arid and semi-arid regions.

## 5 Conclusion

Under conditions of plastic film residue contamination in cotton fields, a C/N ratio of 9:1 significantly reduced the accumulation of plastic film residues across all three irrigation levels, enhanced soil water-holding capacity, reduced salt accumulation, and consequently improved the uniformity of water and salt distribution. Soil moisture content under the C/N = 9:1 treatment was 4.2%–13.7% higher than under other C/N ratio treatments; the moisture uniformity coefficient increased by 1.3%–11.4%; salt content decreased by 0.1%–7.6%; and the salt uniformity coefficient increased by 1.6%–6.7%. Cotton lint yield under a C/N ratio of 6:1 was 1.4%–11.6% higher than that of other C/N treatments. Furthermore, compared to the C/N=0:1 treatment under both light deficit irrigation and full irrigation, the C/N ratio of 6:1 increased soil moisture content by 4.2%, improved moisture uniformity by 3.1%, and reduced salinity by 1.1%. Ultimately, under conditions of 20% water savings, yield was increased by 9.1%. Therefore, adjusting the C/N ratio in fertilization is an effective strategy for achieving high and stable yields and efficient water use in cotton fields contaminated by residual plastic film in arid and water-scarce regions. The results of this study provide important theoretical support and technical references for optimizing water and fertilizer management models for cotton in such regions.

## Data Availability

The original contributions presented in the study are included in the article/supplementary material. Further inquiries can be directed to the corresponding author.
